# Promoting fruit and vegetable consumption during the COVID-19 pandemic – SportStudisMoveYou (SSMY): A randomized controlled trial

**DOI:** 10.3934/publichealth.2022048

**Published:** 2022-10-10

**Authors:** Joram Weber, Claudio R. Nigg

**Affiliations:** Department of Health Science, Institute of Sport Science, University of Bern, Bremgartenstrasse 145, 3012 Bern, Switzerland

**Keywords:** fruit, vegetable, behavior, intervention, health, COVID-19

## Abstract

**Background:**

The stay-at-home circumstances due to the global coronavirus pandemic have had some negative impacts on people's eating behavior.

**Purpose:**

Therefore, this study attempted to improve fruit and vegetable (FV) consumption intention and behavior through an online video intervention based on the social cognitive theory.

**Methods:**

Participants were recruited with a promotion video posted on social media channels. After consenting and completing a pre-survey, participants were randomly assigned to either a) the intervention group addressing FV consumption or the b) attention control group addressing physical activity. After two weeks, the participants completed an online post-survey.

**Results:**

The participants (N = 82) were 77% female and 50% students; 95% chose German for the survey language, and 84% were from Switzerland. The mean baseline FV consumption intention was 3.05 (standard deviation: 1.03), and FV consumption was 4.64 (standard deviation: 2.06) portions a day. The analysis showed no significant change in FV consumption intention (F = (1, 78) = 0.02, p = 0.88, ηp^2^ = 0.00) or behavior (F = (1, 78) = 0.019, p = 0.89, ηp^2^ = 0.03) due to the intervention.

**Conclusions:**

Plausible reasons why no significant effect was found were the brief intervention duration, the setting and insufficient tailoring. The lessons learned from this study are to plan better, tailor more to the needs of participants and carefully consider the goals before promoting an intervention.

## 1. Introduction

The current global coronavirus (COVID-19) pandemic has impacted social [1], economical [2], physical health [3] and mental health [4],[5] aspects in people's lives. Specifically, regarding eating behaviors, the access to markets and restaurants was severely limited or shutdown altogether, and people were only allowed to leave the house for essential shopping, such as buying food or housewares [6]. This changed people's food-related behaviors. Goldman [7] found in an analysis of Google Trends Data that the term “cooking” was more often searched at the end of March in 2020 as compared to the months prior. There was a documented increase in home cooking and baking [8]. Although cooking may be related to healthy eating, not following cooking recipes can lead to overeating [9].

Many countries implemented “stay-at-home” policies, which meant setting up home offices and entertaining oneself at home; consequently, people changed their lifestyle to one that is more in front of a screen [10]. Screen time, even in the absence of food advertising, has been found to be associated with increased dietary intake compared with non-screen behaviors [11]. Thus, the related risk of eating more and unhealthy food likely increased during the stay-at-home period.

Analysis of two Italian cohorts [12] and a cohort from Spain [13] showed that healthy eating improved during the first lockdown. However, the same two Italian cohorts also revealed that psychological distress from the COVID-19 confinement was directly associated with unhealthy dietary modifications [14]. In addition, almost half of another Italian population sample during the first COVID-19 stay-at-home reported an increase in body weight [15]. A longitudinal study from Italy [16] confirmed that obese children and adolescents changed their eating, activity and sleep behaviors to be more unfavorable during the national “stay-at-home” period. The intake of potato chips, red meat and sugary drinks all increased during this time [16]. Rundle et al. [17] also highlighted increased food insecurity for children and the stockpiling of shelf-stable, calorie-dense comfort and ultra-processed foods during the lockdown.

Most ultra-processed foods are very durable, palatable and ready to consume, which is an advantage in a stay-at-home situation. However, ultra-processed products are usually energy dense, have a high glycemic load, are low in dietary fiber and micronutrients and are high in unhealthy types of fats, free sugars and salt [18]. The consumption of energy-dense ultra-processed foods often leads to energy overconsumption, harm to satiety mechanisms and obesity; it is also associated with increased rates of type II diabetes, some cancers, cardiovascular diseases, asthma, gallbladder disease, osteoarthritis and chronic back pain [18],[19].

Fruit and vegetable consumption, on the other hand, is associated with lower risks of type II diabetes [20]–[22], certain cancers [23] and cardiovascular diseases [4],[23]–[25]. Due to the benefits and protective effects of fruits and vegetables, a minimum of 400 g of fruit and vegetable consumption per day is recommended, which is about 3–5 servings [26].

Interventions based on behavioral theory (e.g., social cognitive theory (SCT)) are more effective than interventions that do not apply behavioral theories [11]. According to the SCT, action and motivation are influenced by forethought [27]. SCT factors that influence human behavior are perceived self-efficacy, outcome expectancies, goals, barriers and facilitators. Perceived self-efficacy concerns someone's beliefs in their ability to perform a specific action required to attain a desired outcome. Outcome expectancies are concerned with people's beliefs about the possible consequences of their actions. Individuals weigh the pros and cons of a certain behavior to develop an intention to act or an intention not to act. To adopt a desired behavior, individuals form a goal and then try to execute the action [27]. Goals serve as self-stimulation and lead to health behaviors. They should be as specific as possible and challenging, but realistic, in order to facilitate a desired action. Goal setting also depends on perceived socio-structural factors such as barriers or opportunities of living conditions like health, economic, political or environmental systems [28]. High expectations of self-efficacy, positive outcome expectations, perceived social support and beneficial environmental variables increase the likelihood of changing a behavior [24],[29].

Studies have shown the positive effects of SCT on healthy eating [30],[31], and more specifically, on the consumption of fruit and vegetables [27],[32]. Online interventions based on SCT have also shown positive effects on healthy nutrition [33].

However, no interventions have been found to date that use SCT to address fruit and vegetable consumption during a time of crisis such as the COVID-19 stay-at-home situation. The purpose of this study was to examine the effect a brief SCT-based intervention delivered online on fruit and vegetable consumption intention and behavior during the COVID-19 lockdown period. The following hypotheses were investigated:

1. Effectiveness hypothesis: The intention and consumption of fruits and vegetables will increase in the intervention group, whereas no change is expected in the attention control group.
2. Dose hypothesis: The more often the participants watch the videos, the stronger will be the effect on the intention to eat and the actual consumption of more fruits and vegetables.
3. Moderator hypothesis: Due to the novel nature of the pandemic, an exploratory hypothesis is put forth for the intervention to be moderated according to sex, age and education.

## 2. Materials and methods

### 2.1. Design and procedure

The study was a 2 (group) by 2 (time point) randomized control trial. The participants provided informed consent prior to completing the online pre-survey. At the end of the pre-survey session, the participants were randomly assigned to an intervention (fruit and vegetable (FV)) group or an attention-control (physical activity (PA)) group through a randomization program; at this time, they were also given a link to a YouTube channel containing the five videos for their assigned group. The intervention lasted over a two-week time period during the COVID-19 lockdown. After one week, a reminder email was sent out to the participants containing the link to the five videos of their intervention group again. After two weeks, the participants were emailed a link to complete the post-survey.

### 2.2. Recruitment

The main strategy to recruit participants for the study relied on the online distribution of a short promotion video (1 min 35s). The video's purpose was to raise awareness of the project and motivate people to sign up for it. To obtain a broad sample size (possibly worldwide participation), the spoken language was English with German and French subtitles. In the recruitment phase, the video was shared over a period of nine days through three main promotion strategies. Media Unisport Bern, UNIK Sports, and the influencer Aniya Seki agreed to promote the project. Unisport Bern and Aniya Seki shared SportStudisMoveYou (SSMY) via their social media channels. UNIK Sports shared the project via their social media channels, as well as their newsletter. Another strategy focused on personal contacts. Using a customizable text template, we reached out to as many personal contacts as possible. This was done via WhatsApp, email or personal social networks. Also, project-specific Facebook and Instagram pages were created, where the promotion video and the link to join the project were shared. Lastly, the video was posted on as many Facebook pages as possible. The video was shared on at least 28 different Facebook pages and received over 2500 views.

### 2.3. Measures

#### 2.3.1. Demographics

Sex (male, female, other), age (years), education (years in school) and country were collected in the baseline survey.

#### 2.3.2. Fruit and vegetable consumption and intention

To measure FV consumption, the participants reported the number of servings of fruits and the number of servings of vegetables they consumed daily. One serving was defined as a handful or 120 g, which is about the weight of an average banana or a small apple. These items have documented validity and reliability [34]. Servings for fruits and servings for vegetables were summed for a combined daily FV consumption score.

Also, the participants were asked if they intended to increase FV consumption. The possible answers were “strongly agree”, “agree”, “neither agree or disagree”, “disagree”, “strongly disagree” or “do not want to answer”.

#### 2.3.3. Intervention dose

To assess the dose, the following question was posed: “How many videos regarding health promotion did you watch in the context of this study?” The answer had to be a whole number.

### 2.4. Intervention

#### 2.4.1. Procedures of the intervention

According to the assigned group (FV or PA), the SSMY participants were provided with five videos that aimed to change their health behavior. The five videos followed the same concept and were based on SCT [35]. Each intervention included one basic motivational video and four videos with specific ideas for behavioral change. The purpose of the basic video was to motivate the people to change their behavior, highlight the benefits and set goals for the following four SCT-related aspects: self-efficacy, outcome expectations, pros and cons and goal setting. In the four videos with the specific ideas for behavioral change, the tasks progressed from easy in the first video to somewhat harder in the last video. The text was spoken in English by a professional male speaker. For wider reach, German and French subtitles were incorporated. The basic motivational video lasted about 1 min and 45 s, and the specific behavioral change idea videos each lasted around one minute.

#### 2.4.2. Intervention group: FV consumption

The intervention's goal was to motivate the participants to try to eat one more portion of fruit and/or vegetable a day. The four behavioral change idea videos contained simple and quick-preparation recipes that do not require special skills or ingredients, and they were designed to help the participants achieve their personal FV goal. The first behavioral change idea video recommended the inclusion of fruits in their breakfast by adding them to the cereals or, as an alternative, the blending of fruits into a fruit smoothie. The second behavioral change idea video provided an oven-based vegetable lunch recipe. The third video focused on healthy snacks, such as a sweet and juicy fruit salad or healthy chips. The fourth video showed the preparation of a vitamin-rich vegetable soup for dinner.

#### 2.4.3. Attention-control group: PA

To garner the same amount of attention but not influence FV consumption, this intervention content focused on PA. This intervention's purpose was to motivate people to add 10–20 min of additional PA every day. The four specific ideas for behavioral change videos provided the participants with PA ideas that can be easily implemented at home without any equipment. Each video contained four PAs; among them, one exercise focused on cardiovascular health, and three exercises targeted large muscle groups, namely, the lower body (legs), the back and the front part of the upper body (chest, shoulders and triceps). Body weight exercises were presented, focusing on the core muscles. Furthermore, the participants were given PAs with different levels of difficulty that involved using objects (e.g., backpack with extra weight and bottles) that are likely to be found at home. The motivational process was supported by setting easy goals and promoting self-efficacy aspects in each of the four videos.

### 2.5. Analysis

#### 2.5.1. Power

A power of 0.8 and a medium effect of F = 0.25 between two groups required an overall n = 73 (GPower 3.1.9.7.).

#### 2.5.2. Data cleaning and preparation

The data sets were exported from Limesurvey and merged by using an individual anonymized code for each participant. Out of 166 baseline participants, 81 persons did not complete the follow-up questionnaire. In addition, three people did complete the follow-up questionnaire but provided no useful information. After excluding those participants, 41 participants from the FV intervention group and 41 participants from the PA attention-control group were included for analyses.

Statistical outliers were adjusted as follows: the minimum and maximum ranges were respectively set as the mean ± three standard deviations, resulting in a range of 0–11 servings at both time points. Only one participant had an out-of-range value (14 servings at pretest), which was adjusted to 11 servings of FVs. Missing data were only found for one demographic variable (education); they were deleted pairwise because the amount was minimal (<4%) and appeared completely at random.

### 2.6. Ethics approval of research

This study was conducted according to the guidelines laid down in the Declaration of Helsinki, and all procedures involving research study participants were approved by the University of Bern Faculty of Human Sciences Institutional Review Board (IRB). Written informed consent was obtained from all subjects.

## 3. Results

### 3.1. Sample descriptives

The participants (N = 82) were 77% female and 50% students; the average years of education were 12.1 (standard deviation (SD) = 2.0); 95% chose German for the survey language, and 84% were from Switzerland.

### 3.2. Distribution assumption testing

After adjusting the data for outliers, pretest FV consumption skewness was 0.68 (standard error (SE) = 0.27) and kurtosis was 1.00 (SE = 0.54); at post-test, the FV skewness was −0.65 (SE = 0.27) and the kurtosis was 0.30 (SE = 0.54), which indicated that the data were normally distributed. The pretest intention to increase FV consumption skewness was 0.18 (SE = 0.27), and the kurtosis was 0.39 (SE = 0.3); at post-test, the intention to increase FV skewness was −0.3 (SE = 0.27) and the kurtosis was 0.30 (SE = 0.53), which indicated that the data were also normally distributed.

The analyses (not shown) were repeated using non-parametric analyses; the same conclusions were reached.

### 3.3. Outcome analyses

**Figure 1.**
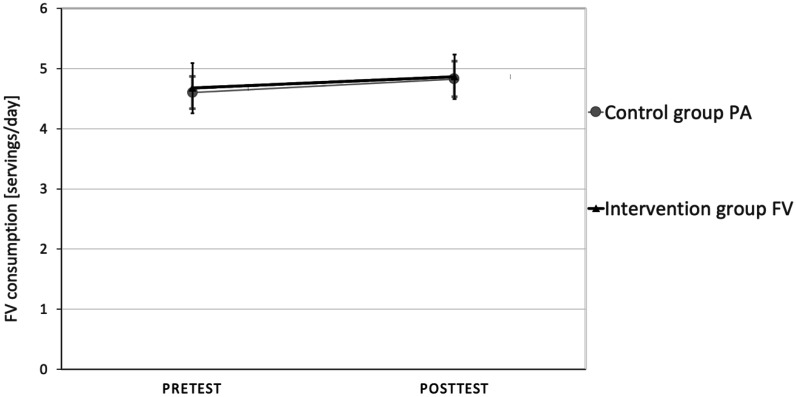
RANOVA results for FV consumption.

**Figure 2.**
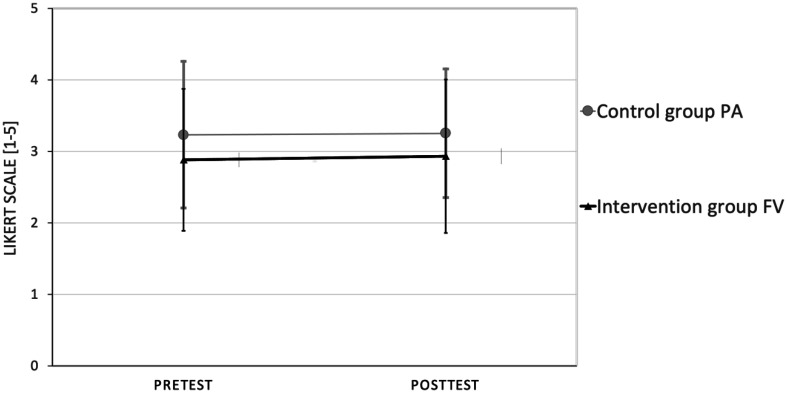
RANOVA results for the intent to increase FV consumption.

The repeated-measures analysis of variance (RANOVA) results revealed a non-significant time by group interaction for FV consumption; specifically, F = (1, 78) = 0.019, p = 0.89 and ηp^2^ = 0.03 (see Figure 1); also, for the intention to increase FV consumption, F = (1, 78) = 0.02, p = 0.88 and ηp^2^ = 0.00 (see Figure 2).

### 3.4. Outcomes regarding meeting FV guidelines

To explore if the intervention had an effect on at-risk participants, which are defined as those not meeting guidelines, the outcomes were compared for the participants who met the FV guidelines at pretest (five servings or more; upper group) and those who did not meet the guidelines at pretest (<5 servings/day; lower group). The RANOVA results showed a significant interaction effect in terms of change in consumption of FV from pretest to post-test in the upper and lower groups F = (1, 78) = 7.00, p = 0.01 and ηp^2^ = 0.08. The lower group increased FV consumption over time, unlike the upper group, which decreased slightly.

Thus, RANOVA was conducted for the upper and lower groups separately within the intervention (FV) and control (PA) groups. No significant interaction was found between the intervention and control groups for the upper intake group (F = (1, 38) = 0.03, p = 0.86, ηp^2^ = 0.077) or the lower intake group (F = (1, 38) = 0.03, p = 0.085, ηp^2^ = 0.001).

The same procedure was conducted for FV intention. The RANOVA results showed no significant interaction for the change in intention of FV consumption from pretest to post-test for the upper and lower groups (F = (1, 77) = 0.19, p = 0.67, ηp^2^ = 0.002).

#### 3.4.1. Dose-response analyses

During the intervention time, the participants watched an average of 4.62 (SD = 2.48) videos. A simple linear regression revealed that the number of times that participants watched the videos did not predict a change in FV consumption (F = (1, 77) = 0.00, p = 0.998, R^2^ = 0.00), nor did it predict a change in the intention of FV consumption (F = (1, 77) = 1.06, p = 0.31, R^2^ = 0.01).

### 3.5. Moderation analyses

The results of analysis of covariance (ANCOVA) tests showed that, in terms of FV consumption, there was no significant time interaction for sex (F = (1, 77) = 1.21, p = 0.27, ηp^2^ = 0.02), age (F = (1, 77) = 0.52, p = 0.47, ηp^2^ = 0.01) or education (F = (1, 74) = 1.23, p = 0.27, ηp^2^ = 0.02).

The ANCOVA results also showed no significant time interaction in terms of the intention of FV consumption for sex (F = (1, 72) = 0.29, p = 0.59, ηp^2^ = 0.004), age (F = (1, 72) = 0.43, p = 0.52, ηp^2^ = 0.006) or education (F = (1, 72) = 0.36, p = 0.55, ηp^2^ = 0.005).

### 3.6. Drop-out analyses

The participants who completed the study were compared to those who did not complete it; no significant difference was found for sex (t(164) = −0.71, p > 0.05), age (t(164) = −0.13, p > 0.05), education (t(160) = −0.35, p > 0.05) or FV consumption at the first measurement time (t(160) = −1.56, p > 0.05). Only the intention to increase FV consumption differed by group (t(159) = 3.17, p < 0.05), as those who completed the study had a lower intention score (mean = 3.06, SD = 1.01) than those who did not complete it (mean = 3.53, SD = 0.87).

## 4. Discussion

Several studies have examined eating behavior and its effects on humans [16],[36],[37], but the studies on interventions to change eating behaviors during the COVID-19 lockdown are lacking. Therefore, the purpose of the SSMY intervention was to increase intention and consumption of FV during the COVID-19 lockdown by using SCT-based online videos. No differences between the intervention and attention-control groups were found. Further, neither sex, age nor education level moderated the effects. This is somewhat surprising, as some studies have shown that women were more likely to suffer psychological stress during and after the recent pandemic, which influenced eating habits [38],[39].

A plausible reason for the absence of any effect of the intervention was the baseline FV consumption, which was already close to the recommended five portions of FV per day. On average, an increase of 0.4 portions of FV per day may not be motivating enough for participants, as they may have already believed that they are close enough to consuming five FV servings a day.

Another reason for the non-effect could be that there may have been intervention contamination, for example, the comparison group may have also accessed the video links of the interventions, as there were no limitations on who could access the other videos. Although the participants were only sent the link to the specific videos, if they searched online for the intervention videos, they would have received access to both types of intervention group videos, because there was no password required to get access. Since it was not assessed which videos the participants watched, the extent of intervention contamination could not be calculated.

Although SCT was used as the basis for the intervention content, it is unknown whether it had an impact, because none of the SCT variables were measured. There was a perceived necessity of a brief measure to minimize the participant burden, as there were no incentives offered. Therefore, another potential reason for the lack of effect is that the intervention was not appropriate or provided at a high enough dose to change the SCT mediator variables. Including theory-based mediator variables is recommended to provide a more in-depth understanding of the mechanisms of health-related behavioral change [40].

Recruitment was performed by Health Promotion Master's students and their professor via social media and direct messages on WhatsApp. The people reached by the recruitment were possibly more aware of health-related topics and have a higher health-related affinity because the reference person is interested in health promotion, which is supported by the relatively high baseline indicators. It is possible that those with no interest or motivation were not reached.

Several limitations need to be considered when interpreting these results. Due to time and space considerations, the FV questions did not include FV drinks or juices, so the results are limited in this regard. The time to prepare the intervention was short because the COVID-19 lockdown in Switzerland lasted only six weeks. This was also the reason why the recruitment time was only nine days and the intervention time was only 14 days, which is quite short to change the intention and behavior of a person. A meta-analysis [41] showed that tailored, interactive and responsive internet programs lead to longer session times per visit and more visits to the website. Therefore, it may be that using only YouTube videos was not sufficiently interactive, tailored or responsive to the needs of the participants. Both groups (attention-control and intervention) were provided access to five videos with the encouragement to watch each video several times; however, the participants watched on average only 4.62 videos over the course of the 2-week intervention. Not watching the videos several times and not even watching all of them on average could have been the reason for the lack of intervention results, since there was no dose-response relation found (the dose was too low).

## 5. Conclusions

This study found no effects of a video-based FV intervention on the intention of FV consumption and actual FV consumption during the COVID-19 lockdown due to various reasons. Therefore, the following recommendations should be considered.

Including a more comprehensive nutrition intervention in terms of food preparation, balanced meals, grocery shopping strategies and cooking is recommended. Future interventions for FV consumption during a pandemic should consider a larger variety of recruitment channels, a longer recruitment time (if possible), individualized tailoring (based on daily FV consumption, sex, age, education, motives, goals and interests) and more interactive and responsive internet material. Suggestions to overcome the barriers of buying fresh FVs during pandemic crises should also be included. Possibly piloting the intervention before conducting the actual intervention to understand what participants perceived to be the main themes of the intervention would be useful for adapting the intervention and maximizing potential effectiveness.

Pandemics like COVID-19 present a higher risk for poor nutrition and unhealthy eating. Research should prioritize developing an effective healthy eating intervention that is appropriate during such a situation to counteract these risks and promote better health.
